# Domain selection combined with improved cloning strategy for high throughput expression of higher eukaryotic proteins

**DOI:** 10.1186/1472-6750-7-45

**Published:** 2007-07-30

**Authors:** Yunjia Chen, Shihong Qiu, Chi-Hao Luan, Ming Luo

**Affiliations:** 1 Department of Microbiology, University of Alabama at Birmingham, Birmingham, Alabama, 35294, USA; 2 Center for Biophysical Sciences and Engineering, University of Alabama at Birmingham, Birmingham, Alabama, 35294, USA

## Abstract

**Background:**

Expression of higher eukaryotic genes as soluble, stable recombinant proteins is still a bottleneck step in biochemical and structural studies of novel proteins today. Correct identification of stable domains/fragments within the open reading frame (ORF), combined with proper cloning strategies, can greatly enhance the success rate when higher eukaryotic proteins are expressed as these domains/fragments. Furthermore, a HTP cloning pipeline incorporated with bioinformatics domain/fragment selection methods will be beneficial to studies of structure and function genomics/proteomics.

**Results:**

With bioinformatics tools, we developed a domain/domain boundary prediction (DDBP) method, which was trained by available experimental data. Combined with an improved cloning strategy, DDBP had been applied to 57 proteins from *C. elegans*. Expression and purification results showed there was a 10-fold increase in terms of obtaining purified proteins. Based on the DDBP method, the improved GATEWAY cloning strategy and a robotic platform, we constructed a high throughput (HTP) cloning pipeline, including PCR primer design, PCR, BP reaction, transformation, plating, colony picking and entry clones extraction, which have been successfully applied to 90 *C. elegans *genes, 88 Brucella genes, and 188 human genes. More than 97% of the targeted genes were obtained as entry clones. This pipeline has a modular design and can adopt different operations for a variety of cloning/expression strategies.

**Conclusion:**

The DDBP method and improved cloning strategy were satisfactory. The cloning pipeline, combined with our recombinant protein HTP expression pipeline and the crystal screening robots, constitutes a complete platform for structure genomics/proteomics. This platform will increase the success rate of purification and crystallization dramatically and promote the further advancement of structure genomics/proteomics.

## Background

One of the results from genome sequencing projects, such as the human genome project, is to promote the development of structural genomics/proteomics endeavors which focus on the large-scale determination of protein structures and functions. The traditional cloning and expression approach is inadequate for such a daunting task, and high throughput (HTP) methods are clearly necessary [1,2]. An integrated robotic pipeline can streamline the complex experimental procedures and makes it possible to carry out gene cloning and protein expression for a large amount of targets in a timely and reproducible manner. Some groups have developed the HTP cloning method including the design of nested primers for PCR cloning [3], while we have also developed an automated pipeline for recombinant protein expression, applying the GATEWAY cloning/expression technology and a stepwise automation strategy on an integrated robotic platform [4]. The robotic pipeline is fully operational and has produced a large number of soluble recombinant proteins in *E. coli *using the open reading frame cDNA library (ORFeome) for *C. elegans *and human genomes [5,6].

However, the success rate of expressing soluble proteins is limited when the full length ORF was used to express the target protein. In a number of cases, including our own results, soluble proteins could be expressed in *E. coli *when a smaller fragment derived from the ORF was used for expression [7-10]. We have identified smaller protein fragments from spontaneous degradation and limited proteolysis, and recloned them for expression [7,8]. Compared to expressing soluble proteins carrying GATEWAY tags due to cloning artifacts, the soluble expression rate was increased from 1.3% to 27.6% when the GATEWAY tags were not included, and a 41.7% rate of soluble expression was achieved when the identified fragment without both GATEWAY tag encoded sequences was recloned (data not shown). The GATEWAY tags named here refer to the amino acid sequences TSLYKKAGX and TQLSCTKW, resulted from the recombination site attB1 or attB2, respectively, generated by the GAETWAY LR reaction [11]. X refers to the amino acid that depends on the coding sequence. With pET15g as the expression vector, which was engineered using pET15b (Novagen) to be compatible with GATEWAY cloning [4], the final N-terminal tag sequences in the originally and newly cloned genes are MGSSHHHHHHSSGLVPRGSQS*TSLYKKAGX *and MGSSHHHHHHSSGLVPRGSQS*TSLYKKAG*LVPRGS respectively, in which HHHHHH is the his-tag followed by a thrombin cleavage site (LVPR|GS, named thrombin site I, the cutting site is between R and G) deprived from pET15b vector, *TSLYKKAG *is the N-terminal GATEWAY tag generated by GATEWAY LR reaction, and the last LVPRGS is the newly introduced thrombin site (named thrombin site II) that is used to eliminate the N-terminal GATEWAY tag. No C-terminal GATEWAY tag was present in the newly cloned genes by the introduction of a stop codon after the coding sequence. Thus the clones in which GATEWAY tags were included expressed a recombinant protein that had Sequence I, i.e. GSQSTSLYKKAGX at the N-terminus and Sequence II, i.e. TQLSCTKW at the C-terminus in addition to the coding sequence after the his-tag was removed by protease digestion through the thrombin site I. In the clones without the GATEWAY tags, the recombinant protein contained only GS at the N-terminus in addition to the coding sequence. More recently, 23 fragments were recloned and 6 of them have resulted in diffracting quality crystals, which led to 3 structures [7,8]. These findings suggested that the sequences derived from GATEWAY tags affect the soluble expression and a well folded fragment/domain of the target protein is best suited for expression of a soluble recombinant protein in *E. coli*. In fact, 90% of the structures of human proteins deposited in the Protein Data Bank (PDB) [12] comprise a fragment of the gene. We therefore modified our robotic pipeline to incorporate an automatic operation that can select a proper domain/fragment from the ORF for recombinant protein expression and used new cloning strategy described above.

New bioinformatics tools and cloning methods were developed and adopted to the previously established robotic pipeline, as discussed in this report. The major modifications included the automatic design of PCR primers, and improved multi-step laddered PCR, followed by previously established micro BP reaction of GATEWAY cloning, transformation, plating of transformed *E. coli *cells (DH5α), colony picking and entry clone plasmid DNA extraction. The automated cloning module is combined with our automated protein expression module that consists of construction of expression clones in 96-well plates, protein solubility profiling by dynamic ELISA, as a protein expression platform for structural genomics/proteomics. The cloning module is flexible and efficient to carry out different cloning strategies as shown here.

A number of algorithms for predicting domain boundaries have been developed previously [13-18]. Most of them, however, are not publicly available or cannot be adapted to our HTP pipeline. We report here a new composite scheme to locate domains with relatively accurate boundaries. Programs included in the scheme are InterPro/InterProScan [19,20] and Domain Linker Finder [16], BLAST [21], SignalP [22,23] and TMHMM [24]. The BLAST alignment and signal peptide, transmembrane (TM) region prediction were combined with the results of InterPro/InterProScan and Domain Linker Finder to define the fragment for cloning. This composite method has been validated with experimental results.

## Results and discussion

### HTP cloning of 366 ORFs

**Figure 1:**
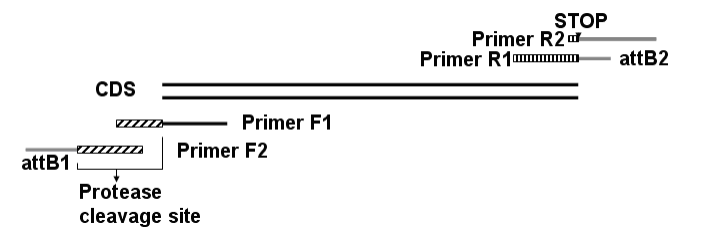
**The primer design strategy using two pairs of primers**. Primer F2 and R2 contained attB sites and no gene specific region, which could be synthesized in bulk; Primer F1 and R1 contained gene specific sequences and an overlap region with Primer F2 and R2. CDS stands for coding sequence and a protease cleavage site was engineered after attB1 site.

The GATEWAY system is a suitable method for HTP cloning in 96-well plates. However, when entry clones (generated with pDONR201) and the expression vector pET15g are combined by the LR reaction, the recombination sequence attB1 may add additional unwanted 9 amino acids (TSLYKKAGX) at the N-terminus if the insert is downstream from a fusion peptide, and the attB2 site may add TQLSCTKW at the C-terminus if no stop codon follows the coding sequence. We named sequences from attB1 and attB2 as the GATEWAY tags. The additional amino acids derived from GATEWAY tags may interfere with subsequent experiments, such as soluble expression of the recombinant protein, purification problems due to aggregation of the protein, and crystallization of the protein (see descriptions in Background). It is therefore desirable to engineer a protease (thrombin here) cleavage site (PCS) after attB1 (Figure 1). A stop codon was also added right after the coding sequence in primer design to eliminate the extra amino acids at the C-terminus due to GATEWAY cloning. After the protein is purified, all amino acids prior to PCS, i.e. MGSSHHHHHHSSGLVPRGSQSTSLYKKAGLVPR, can be removed by the protease cleavage. Compared with the clones in which GATEWAY tags were included, newly cloned and expressed recombinant proteins contained only GS at the N-terminus in addition to the coding sequence. And if no new PCS was introduced, expressed proteins would have Sequence I, i.e. GSQSTSLYKKAGX at the N-terminus and Sequence II, i.e. TQLSCTKW at the C-terminus in addition to the coding sequence after the his-tag was removed by protease digestion through the thrombin site I (For details, see Background). Since the PCS was included in the primer synthesis in our strategy and the long forward primer would be costly and could increase the chance of errors, we designed a PCR strategy using two forward primers and two reverse primers (see Methods: Primer design and the PCR protocol for HTP cloning). This strategy has two advantages: only short primers are required, and primer F2, R2 could be synthesized in bulk. Such measures significantly reduce the cost and the error rate in 96-well operations.

A comprehensive computer program has been developed to carry out primer designs for selected genes. Usually the length of the gene-specific nucleotides in the entire primer should be maintained between 20 to 30 bases according to the manufacturer's manual [25] and our previous experience. The length of gene-specific oligos in this program is therefore set in this range. Since PCR clones are to be carried out in 96-well plates, conditions for all wells, such as denaturation time, cycle number, are the same even though each well represents a different gene. Therefore in addition to grouping coding regions with a similar length in one plate, we also chose to design primers that would result in a similar melting temperature (Tm). The best value for Tm was about 60°C for our experiments, so we tried to make the Tm of all oligos as close to 60°C as possible by adding or subtracting one base at a time. Besides the length of oligos, the salt concentration can also affect the Tm. In our program, the salt concentration was set at 10 mM. After the gene-specific oligo was designed with optimal Tm, sequences corresponding to attB1 or attB2, PCS and a stop codon were added. The primer design program was written in PERL, which could be easily modified to accommodate changes in primer sequences.

**Figure 3:**
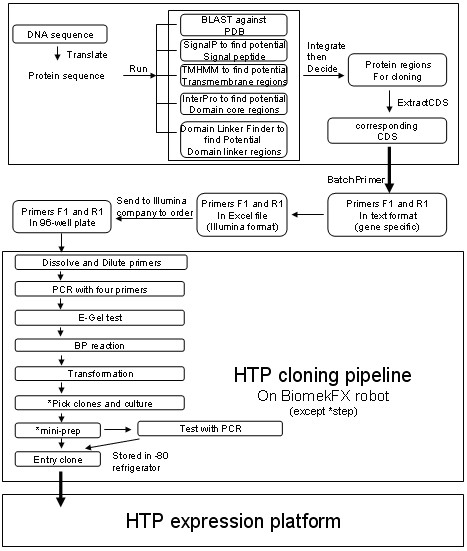
**A schematic representation of HTP cloning and expression pipeline with the aid of bioinformatics tools**. In above HTP cloning pipeline, some steps, which were marked with star, were not performed on BiomekFX robot. ExtractCDS and BatchPrimer were two PERL programs used for extraction of the DNA coding sequence from a full-length sequence (ORF) and design of gene specific primers.

**Figure 4:**
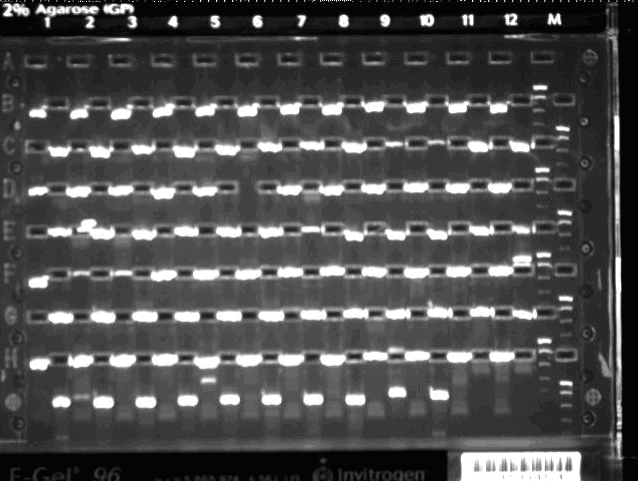
**An E-Gel test result for entry clones of the second plate of 94 human genes**. 2% E-Gel^® ^96 Agarose with E-Gel^® ^Low Range Quantitative DNA Ladder were used in the test.

After receiving primers for 90 *C. elegans*, 88 Brucella, and 188 human ORFs in 96-well plate, HTP cloning (Figure 3), including PCR, E-Gel check, BP reaction, transformation, colony picking, cell culture and mini-prep, was performed on our integrated robotic platform. From 366 attempted amplifications, 337 PCR products could be detected by E-Gel (Figure 4). Interestingly, 20 vectors, out of 29, whose PCR products could not be detected by E-Gel could still be transformed and obtained as entry clones successfully. This phenomenon has also been observed by other research groups [26]. Including clones that were derived from PCR products not detectable by E-Gel, but transformed successfully, our PCR protocol showed a success rate of 97.5%. Our follow-up results suggested that PCR determines the final success rate of the whole HTP cloning process (Table 1), whereas other steps, such as BP reaction, transformation, have negligible effects on the final outcome. Finally 96.7% ORFs were obtained as entry clones, which were verified by PCR/E-Gel check.

**Table 1:** Statistic of PCR and entry clone success rates of HTP cloning

	all	PCR (success rate)	entry clone (success rate)
*C. elegans*	90	85 (94.4%)	83 (92.2%)
Human	188	184 (97.9%)	183 (97.3%)
Brucella	88	88 (100%)	88 (100%)
Total	366	357 (97.5%)	354 (96.7%)

### Validation of domain identification

Proteins are usually composed of multiple domains connected by linkers. Removal of flexible tails or separation of fragments would yield more compact and stable protein fragments that are more suitable for expression of a soluble recombinant protein and subsequent studies including crystallization, as demonstrated by data presented below. We aimed at developing an integrated strategy, named DDBP (domain/domain boundary prediction), to predict domain boundaries and stable fragments within the full length protein coded by the ORF. In this strategy, InterPro/InterProScan, PDB homology alignment, and Domain Linker Finder were the core methods used for domain prediction. In addition, signal peptide prediction by SignalP and TM regions prediction by TMHMM provided supplementary information for more accurate prediction.

InterPro is an integrated database that consists of most of the essential databases for domain and function site available today, such as PFAM [27], ProDom [28], SMART [29], PRINTS [30], PROSITE [31], TIGRFAM [32], SUPERFAMILY [33], etc. InterProScan, which is used together with InterPro database, is a tool that combines different protein signature recognition methods into one resource. Since InterPro contains many different domain and function site databases, conflicted results often appear when different databases were used. Moreover, InterPro/InterProScan analysis could only predict the core region of a domain, but not the domain boundaries. To improve the prediction accuracy, Domain Linker Finder (DLF), which applies the neural network method to distinguish domain linker sequences from non-linker sequences, was used to confirm domain prediction results obtained by InterPro/InterProScan, and to define more accurately the domain boundaries.

As the first step of DDBP, prediction of the signal peptide and the TM region for each ORF was carried out by SignalP and TMHMM, respectively. The identified signal peptide was eliminated as an unstable region, and TM regions would be treated as domain linkers that were later integrated into the results from DLF. The second step is to perform BLAST analysis against the PDB database to find potential domain relevant information. Finally InterPro/InterProScan and DLF programs were executed.

**Figure 5:**
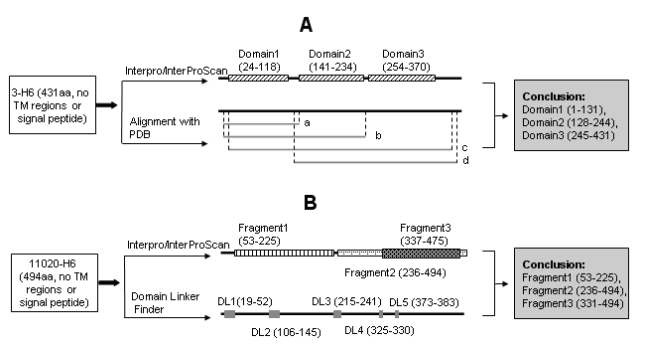
**Two examples for interpreting DDBP (domain/domain boundary prediction) method**. **A: **According to the prediction of Interpro/InterProScan, 3-H6 (NP_508026), a 431-amino-acid protein that has no TM region or the signal peptide, possibly contained three domains: Domain1 (24–118), Domain 2 (141–234), and Domain 3 (254–370). a, b, c, d on the right of horizontal lines mark four separate alignment results between protein 3-H6 and Protein Data Bank (PDB) database. a: the region 4–131 of 3-H6 is homology with the region 20–147 of 1ROU with 60% identity; b: the region 4–244 of 3-H6 was similar to the region 41–280 of 1Q1C with 48% identity; c: the region 7–408 of 3-H6 was similar to the region 24-422 of a 1KTO/A with 40% identity; d: the region 128–428 of 3-H6 was similar to the region 22–330 of 1P5Q/A with 35% identity. By combining the results of Interpro/InterProScan and alignments, three protein fragments (1–131, 128–244, and 245–431) were selected for 3-H6 as stable domains/fragments. **B: **11020-H6 (corresponding to the region 299–792 of protein NP_493412), a 494-amino-acid protein that has no TM regions or the signal peptide, was predicted to have three possible domains/fragments (Fragment1: 53–225; Fragment2: 236–494; Fragment3: 337–475) by InterPro/InterProScan (shown on top). DLF results showed that protein 11020-H6 may contain five possible domain linkers (DL1: 19–52; DL2: 106–145; DL3: 215–241; DL4: 325–330; DL5, 373–383) (shown at the bottom). The stable domains/fragments of 11020-H6 were predicted as 53–225, 236–494 and 331–494 by the DDBP method (shown as the conclusion in the box at right).

When results of InterPro/InterProScan and DLF were available, further analyses were performed: (1) if results of InterPro/InterProScan can be confirmed by PDB alignment results, manually integrate them and decide common domain boundary positions. For example, protein 3-H6, i.e. NP_508026 (Figure 5A), which comprises 431 amino acids, has no signal peptide and TM regions according to the prediction of SignalP and TMHMM. The result of InterPro/InterProScan showed this protein contains three possible domains/fragments: Domain1 (24–118), Domain2 (141–234) and Domain3 (254–370). While PDB alignment results showed: (a) the region 4–131 of 3-H6 is homologous to the region 20–147 of a 149-Amino-acid protein (PDB ID: 1ROU, containing 1 domain) with 60% identity; (b) the region 4–244 of 3-H6 is similar to the region 41–280 of a 280-amino-acid protein (PDB ID: 1Q1C, containing 2 domains) with 48% identity; (c) the region 7–408 of 3-H6 is similar to the region 24–422 of a 457-amino-acid protein (PDB ID: 1KTO/A, containing 3 domains) with 40% identity; (d) the region 128–428 of 3-H6 is similar to the region 22–330 of a 336-amino-acid protein (PDB ID: 1P5Q/A, containing 2 domains) with 35% identity. The results from InterPro/InterProScan prediction appear to be consistent with the results of PDB alignments. By combining these two results, three protein fragments were selected for 3-H6: 1–131, 128–244, and 245–431 as the stable region. (2) if results of InterPro/InterProScan and PDB alignments were not consistent, but one of two results could be confirmed by DLF, the consistent results were manually combined and domain boundary positions were assigned. TM regions were integrated with the result from DLF at this stage as well. For example, 11020-H6, i.e. the region 299–792 of NP_493412 (Figure 5B), a 494-amino-acid protein without TM regions and a signal peptide, was predicted to have three possible domains/fragments by InterPro/InterProScan (Fragment1: 53–225; Fragment2: 236–494; Fragment3: 337–475) and no homologous protein structures were found by PDB alignment. DLF results showed that protein 11020-H6 may contain five possible domain linkers (DL1: 19–52; DL2: 106–145; DL3: 215–241; DL4: 325–330; DL5, 373–383), in which DL1 and DL3 were consistent with Fragment1, the N-terminal end of Fragment2; and DL4 was consistent with the N-terminal end of Fragment3. DL2 was ignored. Since Fragment3 was contained within Fragment2, it is possible that Fragment2 might contain at least two domains, and Fragment3 might be one of them. The final predicted stable domains/fragments of 11020-H6 were: 53–225, 236–494 and 331–494; (3) if results of InterPro/InterProScan and PDB alignments were not consistent, and no result from DLF was available or the DLF prediction didn't support any results from InterPro/InterProScan or PDB alignments, the N-terminus and C-terminus of the ORF would be treated as domain boundaries. After completing the prediction, a final check was performed to ensure that the region between two predicted domain boundaries should be at least 80 amino acids. If a predicted domain contained less than 80 amino acids, one of the two domain boundaries with a less reliability would be omitted and the domain was joined to the next domain/fragment, except that positive PDB alignment results were available and supported that the short predicted domain was long enough to form a stable domain.

In order to validate this combination scheme, we constructed a dataset that contains the definition of 47 domains/fragments from our experimental results (see Method: Datasets for domain/domain boundaries prediction) and made a comparison between the experimental and DDBP prediction results (Table 2). In the comparison, the experimentally determined domain/domain boundaries are assumed to be correct domain/domain boundaries. For a protein, whatever how many domains it contained or were predicted, if only two boundaries of one predicted domain were same with those of one correct domain, or its ranges < = +10 aa, this prediction would be as an accurate prediction. Similarly, if 10 aa < ranges < = +30 aa, the prediction would be as a basically accurate prediction, and if range > +30 aa, the prediction would be as a wrong prediction. For example, protein 11011-D8 (Table 2) has one experimental determined domain: 45–190. With DDBP method, it was predicted with two possible domains: 1–107 or 52–190. Because one of predicted domains (52–190) was consistent with the correct result, i.e. ranges (52-45 = 7 and 190-190 = 0) < = +10 aa, this prediction was as a accurate prediction. Protein 4-F5 (Table 2) has one experimentally determined domain (1–144) and its predicted domains by DDBP method were 1–124 and 143–269. By comparison, 4-F5 was thought as a basically accurate prediction because its ranges (1-1 = 0 and 144-124 = 20) < = +30 and > +10 aa. The complete comparison for all 47 domains were listed in Table 2, as showed that more than 60% of the prediction was consistent with experimental results, in which 43% was accurate (labeled with I in the column A) and 19% was basically accurate (labeled with II in the column A).

**Table 2:** Comparisons between experimental and DDBP prediction results*

**A**		**B**	**C**	**D**	**E**	**F**	**G**	**H**	**I**	**J**	**K**
**I**	11058-C7	249	no	no	4–190; 1–220; 80–190	no	24%, (7–240/8–268, 288); 26%, (4–240/7–225, 251)	(1–249)#	1–249	NP_506406	F20G2.1
**I**	11048-D3	199	no	no	9–199	76–98, 181–181	26%, (5–160/3–164, 208)	(1–199)$	1–199	NP_502315	F35G2.2
**I**	11011-D8	190	no	no	8–36, 47–75, 83–111; 2–107;	148–172, 108–120, 82–92, 49–51	27%, (37–105/1–69, 146)	(45–190)&	1–107, 52–190	NP_493641	F23F1.2
**I**	18-A2	210	no	no	29–79; 108–194	no	30%, (75–193/4–115, 135)	(74–210)&	75–210	NP_491893	BAG1 (human) homolog family member (bag-1)
**I**	11033-F3	208	no	no	6–74; 128–194; 1–97; 80–207	39–56	31%, (2–207/1–197, 198)	(1–208)#	1–208	NP_496863	Glutathione S-Transferase family member (gst-16)
**I**	11-D11	346	no	no	80–317; 55–320; 219–317	19–34, 116–126	31%, (76–334/36–291, 298)	(56–346)&	55–346	NP_491872	C55B7.3
**I**	11104-F4	370	no	no	2–221, 1–370	126–143, 348–352, 19–25, 71–82, 292–297, 233–237	34%, (128–347/15–231, 265)	(125–370, 1–124)&	1–125, 128–347	NP_001040820	Cell Division Cycle related family member (cdc-37)
**I**	79-D4	401	no	no	65–395; 37–400	66–108, 27–45, 108–125, 217–236, 138–146	35%, (212–395/2– 185, 185)	(206–401)#	212–401	NP_491735	C06A5.7b
**I**	9-H3	212	no	no	no	19–52, 162–192, 79–89	35%, (86–136/84– 131, 217)	(59–212)$	53–212	NP_493365	Y40B1B.5
**I**	76-D4	254	no	no	2–171; 3–250; 139–167	139–147	36%, (3–251/8–265, 278)	(1–254)#	1–254	NP_001021765	Y47G6A.22
**I**	8-C1	142	no	no	4–140	no	46%, (5–141/9–149, 150)	(1–142)&	1–142	NP_499813	T12D8.6
**I**	11-F6	327	no	209– 231, 246– 268	207–227, 246–266	56–95, 19–56, 95–136, 136–161	50%, (141–167/1– 28, 163)	(1–182, 1–145)&	1–135	NP_491774	T09B4.5a
**I**	1-F11	229	no	no	148–217; 170–201; 170–212	19–21, 129–134	59%, (135–220/20–107, 113)	(135–229)#	135–229	NP_506367	F53F4.3
**I**	3-H6	431	no	no	24–118, 141–234; 254–370; 261–370	397–413, 214–225, 100–107, 123–128	60%, (4–131/20–147, 149); 48%, (4–244/41–280, 280); 40%, (7–408/24–422, 457); 35%, (128–428/22–330, 336);	(1–135)#	1–131, 128–244, 245–431	NP_508026	FK506-Binding protein family member (fkb-6)
**I**	20-H6	496	no	no	38–496; 186–261, 293–363, 422–483; 183–272, 275–374, 422–487	120–151, 265–279	66%, (394–496/1– 103, 104) ; 75%, (183–269/1– 87, 90); 75%, (290–366/1– 78, 85)	(169–385, 386–496)&	183–272, 290–366, 394–496	NP_001022967	U2AF splicing factor family member (uaf-1)
**I**	1-D10	206	1–21	no	41–198; 23–77, 85–141, 143–196	no	no	(23–206)#	22–206	NP_491320	R12E2.13
**I**	11020-H6**	494	no	no	53–225; 337–475; 336–453; 236–494	19–52, 106–145, 215–241, 373–383, 325–330	no	(1–237, 238–494) &$	53–225, 236–494, 331–494	NP_493412	Y37H9A.3
**I**	70-H8	130	no	107– 129	109–129	19–104	no	(1–130)#	1–130	NP_491052	W03D8.3
**I**	8-C9	183	no	no	125–159;	no	no	(1–183)# ; (28–183, 23–183)$	1–183	NP_510277	BMP receptor Associated protein family member (bra-1)
**I**	11005-B8	245	no	no	no	19–20, 129–136, 41–46	no	(9–245)#	1–245	NP_740981	R05F9.1b
**II**	18-F7	288	no	no	34–286	19–30, 72–78, 196–202, 66–70	45%, (35–286/21– 274, 276)	(32–266)$	34–286	NP_001021584	EXOnuclease family member (exo-3)
**II**	4-F5	592	no	no	189–269	485–509, 125–142, 19–37, 369–382,99–109, 311–317	35%, (210–269/15–74, 76);	(1–144)&	1–124, 143–269	NP_494544	C16C8.16
**II**	11011-C6	162	no	no	4–162; 5–54, 71–132	51–68	no	(1–148, 1–124)&	1–162	NP_500324	F42A6.6
**II**	11058-H2	249	no	no	4–190; 4–230; 4–209	no	40%, (5–245/23– 263, 267)	(1–222)$	1–249	NP_506407	F20G2.2
**II**	76-F10	263	no	no	23–130; 33–109	118–126, 20–20	27%, (27–120/6–98, 108)	(1–129)&	21–130	T26031	hypothetical protein W01A8.2
**II**	79-H11	245	no	no	2–156, 1–241	144–181, 181–204, 121–144	51%, (2–154/2–154, 155)	(1–185)$	1–156	NP_492567	C03D6.5
**II**	25–B11	302	no	no	23–127; 45–114	203–236, 19–24, 236–248, 146–161, 187–195	no	(1–153)&	23–145	NP_492781	B0511.7
**II**	11-D3	313	no	no	28–214; 9–302	151–174, 19–22	no	(1–313)# ; (213–313)$	23–313	NP_001021333	Suppressor of PResenilin defect family member (spr-2)
**II**	11058-F12	272	no	no	40–63; 40–68, 90–124	no	32%, (67–119/3–54, 60); 26%, (40–119/5–84, 87); 31%, (41–114/36– 113, 124)	(1–147)&	1–124	NP_503566	F36F12.8
**III**	20-D7	500	no	no	311–395; 41–278; 283–436	387–417, 288–308, 453–469, 481–482	28%, (342–432/68–153, 289)	(1–500)# ; (298–500, 388–500, 407–500)$	1–287, 283–452	NP_491868	lariat DeBRanching enzyme related family member (dbr-1)
**III**	37-G9	245	no	no	no	206–227, 19–21	no	(1–102, 103–245)&	1–245	NP_507040	F14H3.6
**III**	70-D2	265	1–25	15–37	69–243	223–247, 195–199	22%, (128–264/13–121, 135)	(1–130, 1–174)&	1–265	AAC25860	Hypothetical protein C37C3.3
**III**	2-B6	316	no	no	27–294	225–260, 174–191, 149–163, 296–298, 71–82, 84–84, 219–220	23%, (53–168/46– 180, 201)	(71–294)# ; (104–316)$&	1–295	NP_501422	D2096.8
**III**	10-E5	274	no	no	1–80, 108–190	229–246, 193–213	25%, (17–161/32– 166, 196)	(65–237, 1–74, 75–274)&	1–192	NP_502380	C25G4.6
**III**	3-D2	419	no	no	95–128, 133–166; 93–197; 133–166	196–217, 41–62, 259–275, 341–351, 19–21	26%, (95–239/13– 144, 166); 32%, (99–197/8–95, 118)	(140–309, 290–419)&	93–197, 93–258	NP_495087	C17G10.2
**III**	113-H8	588	no	no	232–342; 241–334	492–515, 19–56, 81–132, 150–185	29%, (234–328/2– 85, 105)	(1–345, 34–313)&	232–342	NP_740981	R05F9.1b
**III**	76-F6	803	no	769– 791	17–250, 558–652; 10–34, 111–148, 382–402	752–767, 19–24, 717–727, 280–281	29%, (62–257/5– 200, 205); 29%, (83–257/1– 175, 181)	(1–181)&	25–257	NP_491008	alpha-CaTuliN (catenin/vinculin related) family member (ctn-1)
**III**	4-A4	569	no	no	164–338, 369–527;187–200, 206–222, 263–279, 305–321, 321–335, 506–527; 263–318, 388–452, 511–568	65–97, 19–38,112–139	32%, (154–567/18–397, 402)	(334–542, 334–501)&; (1–569)#	154–549	NP_495753	associated with RAN (nuclear import/export) function family member (ran-3)
**III**	25-H8	339	no	no	24–84; 24–75	215–276, 111–169, 187–201, 100–109	41%, (22–75/7–61, 70)	(1–152)&	1–99	NP_495652	T09A5.8
**III**	2-H9	356	no	no	22–273; 1–108, 115–297; 13–284	192–215, 303–312, 338	42%, (1–284/1–289, 382)	(1–335, 1–356) $&	1–197	NP_497949	T23F11.1
**III**	18-H1	208	no	no	32–124; 36–113; 24–45, 51–68, 131–145, 164–181, 186–205	171–190, 114–171, 19–40	43%, (41–110/10– 76, 90)	(1–81)&$	32–124	NP_510410	HIStone family member (his-24)
**III**	11049-D6	435	no	no	293–433; 270–433	110–152, 234–260, 175–186, 375–381	no	(1–156)# ; (9–158, 1–119)$&	261–435	NP_001041025	Y41E3.7a
**III**	9-G11	250	no	no	1–194	228–232	no	(35–250)&	1–227	NP_497076	R05H10.1
**III**	10-E1	251	no	no	no	158–199	no	(1–206)&	1–251	NP_496943	W01G7.4
**III**	37-F11	230	no	no	1–230	106–126, 168–182	no	(45–179, 45–230)&	1–230	NP_507024	T10C6.5
**III**	75-A8	228	no	no	no	19–44, 125–135	no	(1–189)$&	1–228	NP_492509	F46A9.1
**III**	11048-E2	262	no	no	33–178; 61–179	183–198	no	(1–262)$	1–182	NP_501337	MEChanosensory abnormality family member (mec-17)

* column A: Prediction accuracy level (**I**, accurate; **II**, basically accurate; **III**, wrong) and Plate-ID, users could query/search the sequence relative information from the SGCE web site [41]; column B: number of amino acids in the ORF; column C: signal peptide prediction results using SignalP; column D: transmembrane region prediction results using TMHMM; column E: domains/fragments from Interpro/InterProScan analysis; column F: domain linker regions predicted by Domain Linker Finder; column G: PDB-alignment results, including the percentage of sequence identity, query/subject sequence start position and end position, and the length of the subject sequence; column H: experimental results from protein crystal/three-dimensional structures (labeled with #), limited proteolysis (labeled with $) or spontaneous degradation (labeled with &); column I: DDBP prediction results; J: Accession Number, user could obtain sequence relative information from National Center for Biotechnology Information (NCBI) [40]; K: Definition of the protein or ACEID for proteins without known functions.

** 11020-H6 is corresponding to the region 299–792 of NP_493412.

### Application of the DDBP method and the improved cloning strategy

We applied the DDBP method and the improved cloning strategy to see if the success rate for obtaining purified soluble recombinant proteins would be greatly improved when the predicted fragments were cloned for expressing recombinant proteins in *E. coli*. The test dataset includes 57 proteins from *C. elegans *ORFeome version 3.1, whose expression/purification data of ORFs using the same expression vector were available from previous experiments. For these 57 proteins, the coding regions corresponding to the DDBP predicted fragments were subjected to HTP cloning, and the expression/purification pipeline, in which 14 ones were shortened constructs.

**Figure 6:**
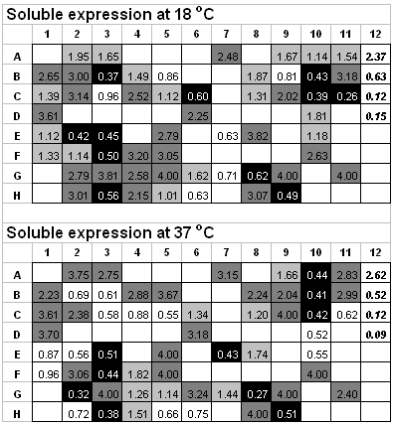
**Soluble expression results of 57 proteins used for testing DDBP method**. ELISA results for soluble expression at 18°C and 37°C. Different shades in panels stand for different expression levels: the dark gray for the higher level, the gray for the medium level, the white for the lower level and the black for those not expressed, which was decided by comparisons with the positive control (A12 and B12, each containing one soluble protein). If ELISA readings of OD (optical density) at 405 nm was higher than or the same with the lower value of positive controls, the protein in this well was considered as expressed. Well C12 and D12 are negative controls and blank wells (white with no numbers) are null. After comparing the results at 18°C and 37°C, seven proteins (well B10, C10, E3, F3, G8, H3, and H9) were considered as not soluble.

**Figure 7:**
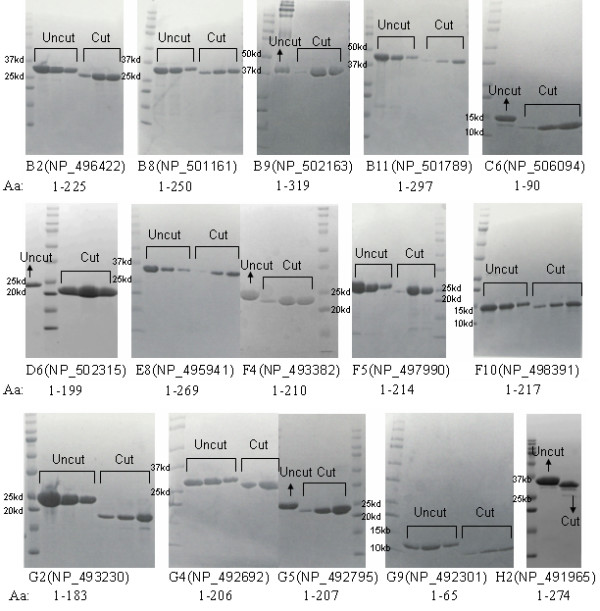
**Purification results of 57 proteins used for testing DDBP method**. Purification results for 15 of the 20 purified proteins. The name of each SDS-PAGE gel includes 2 parts, for example B2 (NP_496422), B2 corresponds to the well showed in Figure 6 and Table 3, and NP_496422 is the accession number of the protein in the public database [40]. The bands labeled with ''Cut'' in the figure correspond to the results after the cleavage by the thrombin and those labeled with ''Uncut'' correspond to the results before the cleavage. ''Aa'' in the figure stands for the amino acid range of the purified proteins.

Previously, all full-length proteins in this dataset, with the GATEWAY tags included at the N-terminus and the C-terminus, were treated as soluble by the 96-well expression profiling when expressed in *E. coli*. However, all but two proteins could not be purified from *E. coli *lysates prepared for expressing these proteins. Most of the recombinant proteins in this dataset were either unstable or formed large aggregates as shown by gel filtration chromatography. In contrast, after employing the DDBP method and improved cloning strategy that avoids GATEWAY encoded sequences, 50 proteins were expressed as soluble (Table 3, Figure 6), and until now, at least 20 were successfully purified (Table 3, Figure 7), among which four proteins had been crystallized (data not shown), despite that seven proteins were insoluble (Table 3, Figure 6). There is a 10-fold increase in terms of obtaining purified proteins from this dataset, as shows the combination of DDBP method and our cloning strategy is successful and results in a clearly improved protein expression and purification. However we do not know whether the observed improvement mainly deprives from a correct domain prediction since most proteins in our testing set only have the shortened or the full length construct and the completely comparison cannot be done.

**Table 3:** Constructs, soluble expression and purification results of 57 proteins used for testing DDBP method

Well									
								
Row	Column	Accession Number	Start position	End position	Length (aa)	Protein definition	Soluble expression level (18°C)	Soluble expression level (37°C)	Purified
A	2	NP_493355	1	300	300	C01A2.5	medium	high	yes
A	3	NP_001022737	1	264	264	X-box Binding Protein homolog family member (xbp-1)	medium	high	not
A	7	NP_498947	1	282	282	PeRoXisome assembly factor family member (prx-19)	high	high	Yes
A	9	NP_497226	1	253	253	W06E11.4	medium	medium	not
A	10	T26925	1	195	195	hypothetical protein Y45F10C.5	medium	not soluble	not
A	11	NP_495146	1	218	218	K05F1.9	medium	high	not
B	1	NP_495062	1	210	210	Helix Loop Helix family member (hlh-26)	high	high	not
B	2	NP_496422	1	225	225	B0491.3	high	low	Yes
B	3	NP_495475	1	197	240	F10E7.2	not soluble	low	not
B	4	NP_496547	21	284	284	W03C9.1	medium	high	not
B	5	NP_496156	29	184	184	R53.8	low	high	not
B	8	NP_501161	1	250	250	F42C5.3	medium	high	Yes
B	9	NP_502163	1	319	319	C10C6.3	high	high	Yes
B	10	NP_500772	55	348	368	ZK354.6	not soluble	not soluble	not
B	11	NP_501789	1	297	297	F25H8.1	high	high	Yes
C	1	NP_501895	1	294	294	R09E10.1	medium	high	Yes
C	2	NP_500890	1	243	243	H32C10.2	high	high	not
C	3	NP_501981	1	388	388	R102.5a	low	low	not
C	4	NP_507039	1	196	196	F14H3.5	high	low	not
C	5	NP_506094	1	183	183	F23H12.3	medium	low	not
C	6	NP_506094	1	90	183	F23H12.3	not soluble	medium	Yes
C	8	NP_505964	20	260	260	T04F3.2	medium	medium	not
C	9	NP_506495	1	252	252	D1086.4	high	high	not
C	10	NP_741113	1	394	419	C32A3.3a	not soluble	not soluble	not
C	11	NP_501199	1	299	299	F55G1.9	not soluble	low	not
D	1	NP_495021	1	197	197	EEED8.12	high	high	not
D	6	NP_502315	1	199	199	F35G2.2	high	high	Yes
D	10	NP_491869	1	232	232	MeDiaTor family member (mdt-18)	medium	low	not
E	1	NP_501936	1	190	190	F01D4.5b	medium	low	not
E	2	NP_506929	26	144	206	F57A10.4	not soluble	low	not
E	3	NP_506929	26	206	206	F57A10.4	not soluble	not soluble	not
E	5	NP_491210	1	249	249	T12F5.1	high	high	not
E	7	NP_506245	35	240	240	R186.3	low	not soluble	not
E	8	NP_495941	1	269	308	T24H10.1	high	medium	Yes
E	10	NP_510298	1	269	269	AMP-Activated Kinase Beta subunit family member (aakb-1)	medium	low	not
F	1	NP_492285	1	239	239	F02E9.5	medium	low	not
F	2	NP_509787	1	195	195	F13E6.1	medium	high	not
F	3	AAZ82857	1	230	230	Hypothetical protein C17H12.13	not soluble	not soluble	not
F	4	NP_493382	1	210	210	Y87G2A.10	high	medium	Yes
F	5	NP_497990	1	214	214	C38D4.9	high	high	Yes
F	10	NP_498391	1	217	217	C56G2.15	high	high	Yes
G	2	NP_493230	1	183	183	W02A11.2	high	not soluble	Yes
G	3	NP_492005	1	189	189	F22D6.2	high	high	Yes
G	4	NP_492692	1	206	206	Y106G6E.4	high	medium	Yes
G	5	NP_492795	1	207	207	C34B2.5	high	medium	Yes
G	6	NP_491736	1	214	214	C06A5.2	medium	high	not
G	7	NP_491358	1	233	233	ZK973.9	low	medium	not
G	8	NP_492301	1	240	240	D1081.9	not soluble	not soluble	not
G	9	NP_492301	1	65	240	D1081.9	high	high	Yes
G	11	NP_491721	1	273	273	B0207.11	high	high	not
H	2	NP_491965	1	274	274	T21G5.4	high	low	Yes
H	3	NP_491348	1	287	287	Y47D9A.2a	not soluble	not soluble	not
H	4	NP_491903	27	330	363	D2092.4	high	medium	not
H	5	NP_496803	1	183	183	F15D4.2	medium	low	not
H	6	NP_491434	1	177	177	C10H11.7	low	low	not
H	8	NP_495527	30	179	179	F45E12.5b	high	high	Yes
H	9	NP_494315	1	276	276	F22E5.8	not soluble	not soluble	not

NP_506094 and NP_492301 are two only proteins with shortened and full length constructs in the test dataset. Notably, the shortened constructs of these two proteins are successfully expressed and purified, while their full length constructs are not soluble or cannot be purified. Though this result has no statistic meaning for DDBP method, it at least affirms that the DDBP is an effective method for some kinds of protein to find proper domain/fragment from the ORF for recombinant protein expression.

## Conclusion

In this paper we presented an effective HTP cloning pipeline and a domain/domain boundary prediction (DDBP) strategy. With this pipeline, four 96-well plates of genes could be cloned into an expression vector in seven days. After integrating the domain/domain boundary prediction strategy, the success rate of purification and crystallization was shown to increase dramatically. Moreover, this cloning pipeline, combined with our recombinant protein HTP expression pipeline and the crystal screening platform, constitutes a complete platform for structure genomics/proteomics. In the next stage, we will improve the accuracy of bioinformatics analysis of domain and domain boundaries and automates all bioinformatics procedures.

## Methods

### Genes for HTP cloning

A total of 90 genes from *C. elegans *ORFeome version 3.1 [5], 188 human genes from Human ORFeome versions 1.1 [6], and 88 genes from Brucella melitensis ORFeome version 1.1 [26] were used for evaluating the automated cloning modules. The cDNAs were provided by Dr. Vidal's group at Harvard Medical School as entry clones.

### Datasets for domain/domain boundaries prediction

Domain definition for 47 proteins was derived from experimental results and the dataset was used for validating the domain/domain boundary prediction scheme. Among them, some domains were defined by protein crystals/three-dimensional structures; some were defined by limited proteolysis or spontaneous degradation (Table 2). The stable fragment from degraded samples was sequenced from the N-terminus and its molecular weight was determined by mass spectrometry. The domain definition was derived from the gene by starting at the N-terminus as sequenced and adding more amino acids in the gene sequence till the molecular weight matched that determined by mass spectrometry. This dataset was used to calibrate the domain/fragment prediction algorithm.

Another dataset that has no relevant experimental information for domain definition was also used to examine this prediction method. This dataset included 57 proteins from *C. elegans *ORFeome version 3.1. Full-length sequences in this dataset have been inserted into expression vectors previously for expressing recombinant proteins in *E. coli *with the GATEWAY tags (data not shown).

### Bioinformatics tools

BLAST [21] was used for alignments between our selected sequences and PDB [12] sequences. InterPro/InterProScan [19,20,36], was used to identify domain/fragment(s) of the ORF selected for generating a stable protein domain/fragment. Domain Linker Finder (DLF) [16,37] was used for finding possible domain linker regions. SignalP [22,23,38] and TMHMM [24,39] were used for prediction of the signal peptide and transmembrane (TM) regions. ExtractCDS, written in PERL, was developed as reported here and was used for extracting proper coding regions corresponding to selected domains. BatchPrimer, a comprehensive primer design program, was also developed here to carry out the batch primer design for the selected sequences.

### Primer design and the PCR protocol for HTP cloning

We designed a PCR strategy of using two forward primers (F1, F2) and two backward primers (R1, R2) (Figure 1), modified from the strategy described by Kagawa and colleagues [34]. Primer F1 contains a part of the protease cleavage site followed by the gene specific sequence of 5'-terminus: CCACGCGGCAGC- 5'gene specific sequence. Primer R1 contains a part of the attB2 site followed by the gene specific sequence of the 3'-terminal: CAAGAAAGCTGGGTTA-3' gene specific sequence. Primer F2 contains the attB1 and the protease cleavage site: GGGGACAAGTTTGTACAAAAAAG CAGGCTTGGTGCCACGCGGCAGC, and R2 contains attB2 and the termination codon: GGGGACCACTTTGTACAAGAAAGCTGGGTTA. Gene specific regions in F1 and R1 are designed by BatchPrimer that would result in a pair of primers with a similar melting temperature (Tm) by adjusting the oligo length. The final Tm calculation was based on the formula of Breslauer and his colleagues [35], in which the salt concentration was set to 10 mM. The length of gene-specific oligos in the program was limited to between 20 to 30 bases according to our previous experimental results.

**Figure 2:**
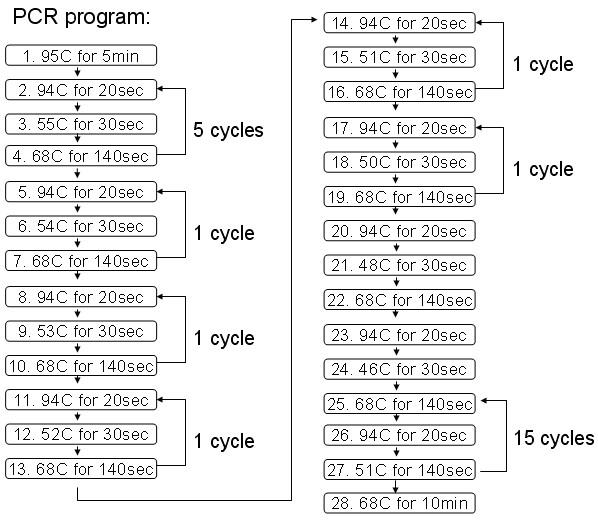
**A multi-step laddered PCR Protocol**. With this protocol, template DNA was amplified for 34 cycles with 5 minutes at 95°C for initial denaturation, 20 second at 94°C for denaturation, 30 second for annealing, 140 second at 68°C for extension and 10 minutes at 68°C for final extension. Annealing temperature was variable: it started from a relatively high temperature (55°C), and then decreased 1–2 degree each time until to 46°C. The temperature again increased 5 degree and stabilized at 51°C.

Different DNA polymerases and different protocols were investigated. After a number of tests, we selected AccuPrime™ Pfx (Invitrogen) as our final choice of DNA polymerase, and a corresponding multi-step laddered PCR protocol was devised as described in Figure 2. PCR starts with primers F1, F2 (F1:F2 = 1:10) and R1, R2 (R1:R2 = 1:10) [34] for 34 cycles. Amounts of oligos, templates and the polymerase are decided according to AccuPrime™ Pfx user manual.

### Gateway cloning and small-scale protein expression

After running the batch PCR protocol, 96-well E-Gel (Invitrogen) was used to check PCR outcomes. Entry clones were generated with entry vector pDONR201 (Invitrogen) and the PCR products by the BP reaction. BP reaction and transformation of DH5α cells were performed according to the GATEWAY protocols from the manufacturer (Invitrogen). Mini-prep was carried out with QIAGEN 96-well mini-prep kits. Expression vectors were prepared in 96-well plates with the selected entry clones and vector pET15g [4], via the LR reaction. Expression vectors were plated, and single colonies were selected for mini-prep. All above procedures (Figure 3), except for colony picking, were automated in our integrated robotic pipeline, operating mainly on a BiomekFX robot, as previously described [4].

For protein expression, expression vectors were transformed into *E. coli *BL21(DE3)AI firstly. Then pick single colonies for recombinant protein expression. After overnight growth at 37°C, the bacteria were diluted (1:200) into 0.6 ml culture containing 100 μg/ml ampicillin in two 96-well block assay plates. After growing for 3–4 hours, without monitoring the absorbance of the culture, protein expression was induced at 18°C and 37°C by addition of IPTG to a final concentration of 1 mM. Protein expression was carried out for 3 hours at 37°C and 20 hours at 18°C.

### Cell lysis and Enzyme-linked Immunosorbent Assay (ELISA)

After protein expression, cells were spun down at 4000 rpm for 30 minutes and cell pellets were lysed by freezing overnight at -80°C and then thawed at room temperature for 15 minutes. Cell lysis was continued by shaking for 30 minutes at 1000 rpm in Vortemp shakers after the addition of 500 μl native lysis buffer (50 mM NaH_2_PO_4_, 300 mM NaCl, 10 mM imidazole, and 1 mg/ml lysozyme, pH 8.0). After lysis, plates were spun at 4000 rpm for 30 minutes and a Beckman Biomek FX robot was used to separate the supernatant, which contained only soluble proteins and was used for the solubility analysis of recombinant proteins by a dynamic indirect enzyme-linked immunosorbent assays (ELISA) protocol, from the pellet.

Indirect ELISAs were carried out on a Beckman/Sagian core system: an ORCA robotic arm (Beckman) for moving plates, a Biomek 2000 (Beckman) for handling liquid, a Biotek plate washer (Bio-Tex Instruments) for washing plates, and a SpectraMax plate reader (Molecular Devices) for recording and analyzing results. A mouse anti-His tag antibody (Anti-Penta-His, QIAGEN) was used as the primary antibody at a dilution of 1:500 and a rabbit anti-mouse IgG Fc alkaline phosphatase conjugate (Pierce) was used as the secondary antibody also at a dilution of 1:500. *p*-Nitrophenyl phosphate (ICN) was used to stain according to the manufacturer's instructions. After read absorbance at 405 nm for 6 hours, with an interval of 30 minutes, the results were electronically compiled and automatically scored with in-house software.

### Large scale expression/purification of soluble proteins and thrombin cleavage of purified proteins

Based on results of ELISA, we performed large scale expression on the possible soluble proteins with same protocols as described above, except enlarging the culture volume from 0.6 ml to 6 liters and inducing cells when absorbance values at 595 nm reached 0.6 to 0.8. After the appropriate incubation (3 hours at 37°C or 20 hours at 18°C), cells were harvested by centrifugation (7000 rpm for 12 minutes). Cell pellets were then re-suspended in appropriate amount of binding buffer (for Ni-His6 affinity column, 20 mM Tris, 500 mM NaCl, 5 mM imidazole, and 0.01% NaAzide, pH 7.9) and completely lysed by sonicating. After centrifuge lysate for 30 minutes at 17000 rpm, remove the pellet and filter lysate through Watmann paper.

Collected proteins were firstly purified by use of the Ni-nitrilotriacetic acid agarose (Qiagen) affinity chromatography: the protein mixture was loaded to the column, and after washed the column, the proteins were eluted under native conditions (500 mM imidazole, 20 mM Tris, 500 mM NaCl, 0.01% NaAzide, pH7.9). Obtained proteins were then concentrated, and further purified by use of the standard protocols with ion-exchange (Hitrap Q column, Amersham) and size exclusion chromatography (superdex75 or superdex200 column, Amersham). Purified proteins will finally be treated with thrombin (Sigma).

For any purified proteins, before treatment with thrombin, a small amount of them were used for optimizing thrombin cutting concentrations: at room temperature, proteins were digested at a series of thrombin concentrations (0.1, 0.5, 1, and 5 unit per milligram of target protein) for 16 hours, and the concentration with the best result was chosen as the actual one. If digestion results were not good enough, try to increase or degrease the amount of thrombin and test again. Once the thrombin concentration was decided, the purified protein was mixed with proper amounts of thrombin and dialyzed in low salt buffer (20 mM Tris, 100 mM NaCl, pH7.5) at 4°C for 16 hours. Resulted proteins were checked by Sodium Dodecyl Sulfate Polyacrylamide Gel Electrophoresis (SDS-PAGE) and used in crystallization trials.

## Authors' contributions

YC developed programs for primer design and sequence extraction (BatchPrimer and ExtractCDS), devised the multi-step laddered PCR protocol, developed the domain/fragment selection method, carried out most gene cloning and protein expression experiments and drafted the manuscript. SQ participated in gene cloning and protein expression experiments. CL participated in gene cloning and protein expression experiments and automated the HTP cloning pipeline. ML conceived of the study, and participated in its design and coordination and helped to write the manuscript. All authors read and approved the final manuscript.
